# Breaking the paradigm—*Prototheca* algae occur only sporadically in soils under a temperate climate

**DOI:** 10.1128/aem.00946-25

**Published:** 2025-07-07

**Authors:** Mateusz Iskra, Mariusz Dyląg, Filip Paluch, Piotr Szwarczewski, Henryk Krukowski, Hanna Bis-Wencel, Jacek Bielecki, Tomasz Jagielski

**Affiliations:** 1 Department of Medical Microbiology, Institute of Microbiology, Faculty of Biology, University of Warsaw 49605, Warsaw, Poland; 2 Faculty of Geography and Regional Studies, University of Warsaw 49605, Warsaw, Poland; 3 Department of Animal and Environmental Hygiene, University of Life Sciences in Lublin 49590, Lublin, Poland; University of Nebraska-Lincoln, Lincoln, Nebraska, USA

**Keywords:** *Prototheca*, algae, soil, occurrence, Poland, temperate climate, humidity, pH, microelements, molecular typing

## Abstract

*Prototheca* are unicellular, achlorophyllous, yeast-like microalgae that have been implicated as the causative agents of opportunistic infections in animals and humans, collectively referred to as protothecosis. Still, the algae are typically saprophytic in nature and mostly occur in areas of high humidity and high organic matter content. Only a few recent studies have explored the environmental reservoir of *Prototheca*. This work is the first study since the early 1980s to investigate the prevalence of *Prototheca* spp. in natural terrestrial sources. A total of 226 soil samples were collected, with only 10 yielding *Prototheca* growth. The overall isolation rate of the algae was 4.4%, with the predominant species being *Prototheca bovis*. These results show that *Prototheca* algae only sporadically occur in soils of the temperate climatic zone. Consequently, this work contradicts the ubiquity of *Prototheca* in soils, breaking the paradigm that, despite the lack of serious experimental appraisal, has long been perpetuated in the literature.

**IMPORTANCE:**

There is a scarcity of studies exploring the environmental habitat of *Prototheca*, a rare and poorly studied genus of microalgae, comprising opportunistic pathogens of humans and animals. This work focuses on the occurrence of *Prototheca* algae in soil environments. Given the potential hazard to human and animal health from exposure to environmental pathogens such as *Prototheca*, the prevalence of the microalgae in soils deserves an insightful examination. The study is the first since the 1980s to assess the prevalence of *Prototheca* spp. in natural terrestrial sources and the first ever performed to approach this topic by molecular methods. Soils have been considered to be ubiquitously inhabited by *Prototheca*. This view has been perpetuated in the literature despite the lack of any serious experimental appraisal. Surprisingly, we found that the algae are very rare soil inhabitants, clearly breaking the prevailing paradigm of their ubiquity in this environment.

## INTRODUCTION

The *Prototheca* genus (Trebouxiophyceae) comprises unicellular, non-photosynthetic, saprophytic, and opportunistically pathogenic microalgae, originally described by Krüger in 1894 (1, 2). The organisms were first recovered from the slime flux of deciduous trees, including elm, locust, and lime (1–4). The algae have also been isolated from other plant sources, such as the bark of a cherry tree, the fruit coat of a loquat, cut stems of banana plants, and potato epidermis (5–8). The first comprehensive study investigating the environmental reservoir of *Prototheca* spp. was performed in the early 1980s, where several ecological niches, including aquatic habitats, soil, and plant material, were searched for the presence of *Prototheca* algae, indicating that they are ubiquitously distributed in nature, with a special predilection for humid and organic-rich environments (9). Since that study, despite the lack of any subsequent investigations in this area, the notion of *Prototheca* ubiquity has been taken as paradigmatic in the literature until very recently, when the problem of the environmental occurrence of *Prototheca* algae has been approached again (10, 11), with a new armamentarium of experimental methods, allowing for much more sensitive and accurate detection of algae (12). Most of these recent works have focused on dairy herd environments since bovine mastitis represents the most prevalent manifestation of *Prototheca* disease in animals (11, 13). The algae have been isolated from the immediate surroundings of dairy cows, specifically from cattle drinking troughs, feeders, bedding material, and milking utensils, with an isolation rate ranging from 9% to 21% (11, 13, 14). More recently, the occurrence of *Prototheca* spp. in water environments has been reassessed, discovering the algae in various water sources, including streams, rivers, lakes, and artificial reservoirs, such as irrigation canals, wastewater effluent, and water treatment facilities, yet at a relatively low overall frequency of 14% (10).

Throughout the already sparse literature on the environmental prevalence of *Prototheca* algae, semi-aquatic and terrestrial habitats have been far less investigated than aquatic ones. Although soils, immediately following water sources, are mentioned as the most common habitat for *Prototheca* spp., there has not been a single monographic study dedicated to the occurrence of algae in terrestrial environments. The only scant data available come from a handful of reports published over the last five decades, mostly describing the dairy farm environment (11, 13, 14). The algae have thus been found in soils on dairy farms in Poland (11, 13), pasture in Haiti (9), stone chambers in Japan (15), and forests in Thailand (16). Other “quasi-soil” sources that yielded *Prototheca* spp. included mud, stream, and riverbank sediments (9, 10). *Prototheca* spp. have also been sporadically isolated from mineral petroleum-contaminated soil (17) and rice paddy fields (9). In the epidemiological context, studies on the ecology of *Prototheca* algae are crucial to assess and counter the risks associated with the contraction and transmission of protothecal disease in both humans and animals. So far, skin contact or oral exposure to *Prototheca* spp. (e.g., wound contamination, ingestion of soil, and drinking water or milk) has been demonstrated as a portal of *Prototheca* infection (18, 19).

This study was undertaken to address a notable paucity of data on the occurrence of *Prototheca* algae in soils. For the first time, the importance of the soil environment as a reservoir of *Prototheca* is evaluated.

## MATERIALS AND METHODS

### Sampling and physicochemical analyses

**Fig 1:**
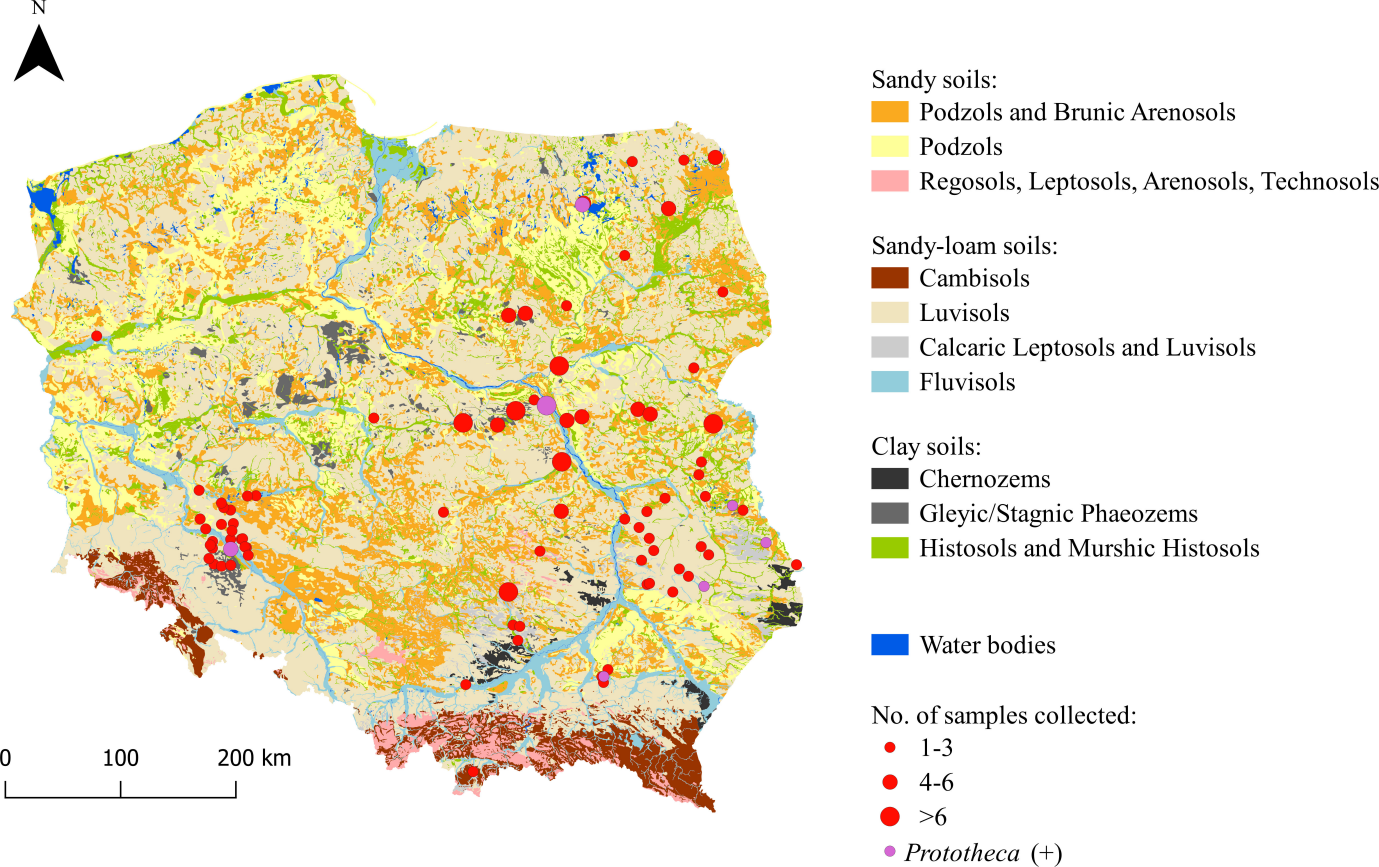
Map of Poland with localities investigated along with sampling sites positive for *Prototheca* spp. in culture. This map was prepared according to IUSS Working Group WRB: World Reference Base for Soil Resources 2014 (20).

This study was performed over an 18 month period (i.e., from April 2021 to October 2022). A total of 226 samples were collected from 140 sampling sites, spanning 83 localities across Poland. The choice of sampling sites was dictated by the maximum possible diversity of climate within the country. The sampling areas were divided into two major profiles. The west-east profile represented increasing continentalism and increasingly harsh conditions toward the east, where the annual amplitude of temperature increases and the precipitation intensity decreases. Whereas the north-south profile reflected changes in the average annual temperature (decreasing toward the north) and the length of the growing season. The precipitation levels were influenced by local topographical features, with reduced amounts observed in regions situated at lower elevations above sea level (lowlands) and marginally elevated levels in exposed terrains, such as lake districts. The distribution of sampling localities in this study is shown in Fig. 1.

Soil samples were collected using a plastic spatula to a depth of 10 cm below ground level. The samples, at least 200 g from each site, were placed into zip bags and delivered within 24 h to the laboratory for microbiological and physicochemical analyses to avoid possible anaerobic conditions. The samples were mixed mechanically by rotating. They were dried and ground prior to detailed analyses. Larger impurities, such as roots and stones, were removed.

The content of available forms of potassium and phosphorus was determined using the Egner-Riehm method (21), which involves the use of lactic acid buffered with calcium lactate at a pH of 3.55. The amount of magnesium was assessed using the Schachstchabel method (22), with the extraction solution comprising 0.0125 M calcium chloride and a soil to extraction solution ratio of 1:10 (wt/vol). Total nitrogen analysis was carried out using the Kjeldahl method (23). Organic carbon was determined by oxidation of potassium dichromate (VI) in sulfuric acid (VI) (24) in accordance with Polish Standard-State Sanitary Institute 14235:2003 (PN-ISP 14235:2003), which is the equivalent of the international standard ISO 14235 (25). The pH and conductivity values were measured using Elmetron equipment (Elmetron, Poland). The conductivity values (in mS/cm) were converted to total dissolved solid (TDS) concentrations (in ppm), which then served to estimate %NaCl, applying the standard conversion: %NaCl = TDS (ppm)/10,000 (26).

### *Prototheca* isolation and culturing

One gram of each soil sample was suspended in 9 mL of liquid *Prototheca* isolation medium (PIM) (27) and pre-incubated at 30°C for 48 h. Subsequently, an aliquot of 0.1 mL of the inoculated sample was spread on PIM agar plates and incubated at 30°C for up to 72 h. Each sample was cultured in triplicate. Colonies suspected to be *Prototheca* spp. were subcultured on Sabouraud dextrose agar (SDA) and subjected to generic identification based on macro- and micromorphology. Wet mount preparations were visualized on a Primo Star light microscope (Zeiss, Germany).

### DNA isolation and molecular typing

Species-level identification was achieved by molecular typing, with the mitochondrially encoded *cytb* gene as a marker (12). Genomic DNA isolation was performed as described elsewhere (28). Briefly, a loopful of cells from SDA plates was suspended in β-mercaptoethanol-lyticase-proteinase K-containing buffer in a tube with 1–2 mm glass beads. The suspension was vortexed vigorously and centrifuged, and the aqueous phase was transferred to a fresh tube prior to DNA extraction using a GeneMATRIX Environmental DNA and RNA Purification Kit (EURx, Poland), as per the manufacturer’s instructions. The purified DNA, dissolved in TE buffer (10 mM Tris-HCl and 1 mM EDTA, pH 8.0), was quantified with a NanoDrop ND-1000 spectrophotometer (Thermo Fisher Scientific, USA) and used as a template for PCR amplification or stored at −20°C until use.

Molecular typing involved PCR amplification and PCR restriction fragment length polymorphism (PCR-RFLP) analysis. In short, the partial *cytb* gene (ca. 650 bp) was PCR-amplified with primers cytb-F1 (GyGTwGAACAyATTATGAGAG) and cytb-R2 (wACCCATAArAArTACCATTCWGG), corresponding to positions 197–217 and 820–843 of the *cytb* gene, respectively, in 30 µL PCR mixtures containing 0.2 µM of each primer, ca. 10 ng of template DNA, and 0.5 U of OptiTaq DNA polymerase (EURx, Poland). Thermocycling conditions were 3 min at 95°C, followed by 35 cycles of 30 s at 95°C, 30 s at 50°C, and 30 s at 72°C, with a final extension of 5 min at 72°C (12). The PCR products were visualized on 1% agarose gel stained with ethidium bromide (EtBr) and double-digested with FastDigest enzymes RsaI and TaiI (Thermo Fisher Scientific, USA). The restriction reaction mixtures contained 1.5 µL of FastDigest buffer, 10 µL of PCR product, and 0.5 µL of each enzyme for a final volume of 15 µL. Digestion was performed for 5 min at 37°C, followed by another 5 min at 65°C. The restriction products were separated on 4% (wt/vol) agarose-TBE gels and visualized using EtBr staining. The electropherograms were analyzed with the UVP BioDoc-IT imaging system (Analityk Jena, Germany). An additional restriction with MfeI was performed to further distinguish between *Prototheca cookei* and *Prototheca pringsheimii*. MfeI digestion was carried out under the same conditions as for RsaI/TaiI double digestion.

For confirmatory purposes, *cytb*-based sequencing was performed in parallel to PCR-RFLP identification. The *cytb* amplicons were purified using a Short DNA Clean-up Purification Kit (A&A Biotechnology, Poland) and subjected to Sanger sequencing. Single-strand reads were assembled into a consensus sequence using Clone Manager software version 9.0 (Sci-Ed Software, USA). The assembled sequences were then compared with those available in the Prototheca-ID database (29). Strains were classified as a given species when their sequence similarity was ≥98%.

### Statistical analysis

Statistical analyses were conducted using the IBM SPSS software package (IBM Corporation, USA). Detailed Fisher’s exact tests and Mann-Whitney *U* tests were performed. The significance level was set at the conventional threshold of *P* = 0.05.

## RESULTS AND DISCUSSION

Soil is a complex environment with a diverse microbial community. It has been estimated that a single gram of soil can host up to 10^10^ bacterial cells and up to 5 × 10^4^ distinct species (30), the majority of which remain unknown (31). Fungal abundance has been estimated to be about 2 × 10^6^ cells/g with its biomass ranging from 100 to 1,500 g/m^2^ of soil (32). Algae have been recognized as a significant part of the soil microbial community since the late 19th century (33). The same one gram of soil contains an average of 5.5 × 10⁶ cells of different algal species, which are thus an integral component of the soil microbiome (34). Although Trebouxiophyceans are among the predominant members of algal communities of the soil crust (35, 36), those of the genus *Prototheca* have only anecdotally been reported. This work is the first to look into the occurrence of *Prototheca* microalgae in soils with robust, large-scale sampling. Of the 226 samples collected, only 10 yielded growth of *Prototheca* algae. This translated into the isolation rate of 4.4%. This percentage almost equals that found in a study from one region of Poland (4.2%) (13) but is far distant (18.6%) from what was found in a similar study performed at a national level (11). Likewise, the isolation rate in this work is much lower compared with the findings from Thailand (8.3%) (16) and the United States (16.7%) (9). In all these studies, however, soil samples were taken almost exclusively from the dairy farm environment (9, 11, 13). Differences between the studies in the *Prototheca* isolation rates may reflect differences in the culture protocols applied. There is no standardized method for the isolation and cultivation of *Prototheca* algae from the environment. Furthermore, the low recovery of *Prototheca* algae from soils may, to some extent, relate to the fact that the culture-based approach, as used in this study, may not detect *Prototheca* cells in a viable but non-culturable (VBNC) state. Although there is no direct experimental evidence demonstrating that *Prototheca* species can enter the VBNC state, this possibility cannot be excluded. As with all studies utilizing culture-based methodologies, the present one also incurs a limitation in that the results reflect only the culturable fraction of the *Prototheca* population and may underestimate the total viable *Prototheca* in soils.

In our study, *Prototheca* spp. were detected only in soil samples collected from areas with shallow groundwater levels, located near streams or water reservoirs. The soils in these locations were characterized by periodic reducing conditions, with temporary surface water accumulation. Under these conditions, microorganisms quickly deplete oxygen, creating anaerobic environments that promote the reduction of compounds like nitrates, manganese, and iron (37). All positive samples originated from areas that were neither cultivated nor fertilized, typically representing pastures or fallow lands where herbaceous vegetation was left unmown and grazed by animals. In contrast, *Prototheca* spp. were consistently undetected in hay meadows or arable fields fertilized with manure or other natural fertilizers and frequented by wild animals. Noteworthy, half of the *Prototheca*-positive samples originated from the Lublin province (2.2%; 5/226) (Table 1), where high-quality, organic-rich soils clearly predominate (38). It should be noted that the sampling distribution in the study was uneven across different regions and sampling sites, which could potentially introduce bias into the observed correlations. *Prototheca bovis* was mainly isolated from land-based habitats like pastures and farm sites, often in the vicinity of animals and away from water sources, suggesting adaptation to organic-rich soils. Quite oppositely, *P. pringsheimii*, *Prototheca cerasi*, and *P. cookei* were found in periodically flooded areas with higher conductivity and no direct animal presence, indicating ecological separation. While *P. bovis* preferred animal-impacted soils, other species showed a preference for wetter environments (Table 2). This varies from what we observed in our earlier aquatic study, where *Prototheca wickerhamii* was the most common species (33%), and *P. bovis* accounted for three times fewer isolates (10). In soils, it was *P. bovi*s that clearly predominated over *P. wickerhamii* (46% vs 8%). This indicates the predilection of different *Prototheca* species to occupy different ecological niches, but further studies are needed to confirm these observations.

**TABLE 1:** Distribution of *Prototheca* spp. in soil samples with respect to sampling site characteristics

Variable	No. (%) of samples^*a*^	
	*Prototheca* spp. (+)	Total
Province		
Lower Silesia	1 (0.4/2.9)	34 (15)
Lublin	5 (2.2/13.2)	38 (16.8)
Lubuskie	0 (–)	3 (1.3)
Łódź	0 (–)	9 (4)
Lesser Poland	0 (–)	4 (1.8)
Mazovia	2 (0.9/2.2)	92 (40.7)
Subcarpathia	1 (0.4/25)	4 (1.8)
Podlaskie	0 (–)	17 (7.5)
Świętokrzyskie	0 (–)	17 (7.5)
Warmia-Masuria	1 (0.4/16.7)	6 (2.7)
Greater Poland	0 (–)	2 (0.9)
Sampling site		
Forest	0 (−)	24 (10.6)
Meadow	0 (–)	27 (12)
Pasture	4 (1.8/15.4)	26 (11.5)
Field	0 (–)	61 (27)
Drainage ditch	1 (0.4/4.2)	24 (10.6)
Watercourse^*b*^	2 (0.9/10.5)	19 (8.4)
Water reservoir^*b*^	2 (0.9/10)	20 (8.9)
Farm buildings	1 (0.4/6.7)	15 (6.6)
Other	0 (–)	10 (4.4)
Distance from buildings		
<1,000 m	10 (4.4/5.2)	193 (85.3)
>1,000 m	0 (–)	27 (12)
No data	0 (–)	6 (2.7)
Liming		
Yes	1 (0.4/5.6)	18 (7.9)
No	5 (2.2/2.8)	179 (79.2)
No data	4 (1.8/13.8)	29 (12.9)
Presence of animals^*c*^		
Yes	6 (2.6/3.8)	160 (70.8)
No	0 (–)	38 (16.8)
No data	4 (1.8/14.3)	28 (12.4)
Nearby reservoir/watercourse		
Yes	6 (2.6/5.3)	114 (50.4)
No	4 (1.8/3.6)	112 (49.6)

^*a*^ Number of samples positive (+) for *Prototheca* spp. in culture and total number of samples collected with respect to a specific variable. In brackets are given percentages calculated in relation to all samples for each variable or all samples collected in the study (*n* = 226); –, no algae detected.

^*b*^ Sediment or soil taken from banks.

^*c*^ Applies to both farm and wild animals.

**TABLE 2:** Details of samples positive for *Prototheca* spp. in culture

Province	Sample^*a*^	Distance from buildings (m)^*b*^	Animals^*c*^	Water body nearby^*c*^	pH	*P* (mg/kg)	K (mg/kg)	Mg (mg/kg)	Organic carbon (g/kg)	Total nitrogen (g/kg)	Conductivity (mS/cm)	Species	GenBank acc. no.
Lublin	Pasture	50	+	−	7.4	165	501	180	19.3	2.2	0.38	*P. bovis*	PQ775131
Lublin	Pasture	300	+	−	5.5	71	133	37	10.3	1.1	0.35	*P. bovis*	PQ775132
Lublin	Pasture	300	+	−	7.6	131	79	22	16.8	1.8	0.48	*P. bovis*	PQ775133
Lower Silesia	Drainage ditch	100	+	+	5.9	482	>1,000	>1,000	56.2	3.5	1.74	*P. bovis*	PQ775134
Warmia-Masuria	Water reservoir	0	−	+	7.5	31	12	4.4	1.4	0.2	0.25	*P. wickerhamii*, *P. pringsheimii*	PQ775137, PQ775138
Subcarpathia	Watercourse	0	−	+	7.7	125	100	72	6.3	1.7	0.02	*P. cookei*	PQ775139
Masovia	Watercourse	0	−	+	7.9	136	92	62	4.1	0.7	0.27	*P. pringsheimii*, *Prototheca ciferrii*	PQ775140 PQ775141
Masovia	Water reservoir	75	−	+	7.7	135	124	107	35.9	2.8	0.92	*P. cerasi,* *P. pringsheimii*	PQ775142, PQ775143
Lublin	Farm buildings	0	+	−	7.1	>1,000	>1,000	>1,000	12.5	6.7	ND^*d*^	*P. bovis*	PQ775135
Lublin	Pasture	50	+	+	6.9	>1,000	>1,000	>1,000	11.4	5.8	ND	*P. bovis*	PQ775136

^*a*^ Watercourse/water reservoir, sediment, or soil taken from the banks.

^*b*^ Distance of the sampling site to buildings; 0 marks a distance of less than 1 m.

^*c*^ Presence (+) or absence (–) of animals or water body nearby.

^*d*^ ND, not determined.

A number of physicochemical parameters, such as humidity, pH, salinity, and organic matter content, impact the abundance and diversity of soil algae (39). Although most of the chlorophytan species exhibit optimal growth at neutral pH, some species of this clade display a broader pH tolerance, thriving in acidic conditions (4.8 or less) (40). The availability of organic matter is a critical factor influencing the growth of green algae under heterotrophic conditions. In general, the higher the concentration of organic matter in the environment, the more efficient is the proliferation of algae (41). Moreover, it has been reported that higher salinity reduces microbial activity, microbial biomass, and changes microbial community structure (42, 43).

*Prototheca* algae exhibit considerable tolerance to fluctuations in environmental variables, including pH, salinity, and temperature. They are known for their wide pH range (3–12), as well as for the ability to survive and proliferate under different salinity levels, reaching, for some species, up to 18% (44). The mean pH value of *Prototheca*-positive samples was higher than that of the *Prototheca*-negative samples (7.3 vs 6.4; *P* = 0.04). Likewise, the elevated conductivity, which is a proxy for salinity, was correlated with the presence of *Prototheca* algae (*P* < 0.001). Saline soils may promote growth of *Prototheca* algae by significantly reducing competition for resources from bacterial and fungal communities. Salinity, indicated by a salt content even at low levels (0.1% NaCl), inhibits the development of these communities and shifts their composition and structure (45).

However, some studies suggest that the structure of soil algae communities is shaped more by soil management practices than physicochemical factors alone (46). Land management is closely linked to anthropogenic activities, such as ploughing, crop rotation, fertilization, plant protection treatment, irrigation, and drainage. Intense human activities, such as farming and the use of fertilizers, enhance microbial biomass (47) and richness (36), while pesticides reduce soil algae diversity (48). The dynamic changes in soil potentially provide favorable conditions for species that are more resistant to low pH, salinity, humidity, and physicochemical alterations (47). The majority of *Prototheca*-positive samples (6/10; 60%) were collected from sampling sites clearly associated with human activity (pasture, farm buildings, and drainage ditch) (*P* = 0.011). The likelihood of algal presence in the soil appeared to increase with proximity (<300 m) to residential, industrial, and agricultural buildings (*P* = 0.008).

Unexpectedly, the mean levels of elements (potassium, magnesium, organic carbon, and total nitrogen) in samples positive for *Prototheca* spp. were slightly lower compared to *Prototheca*-negative samples (data not shown). Of the examined elements, only phosphorus exhibited a positive correlation with the presence of *Prototheca* algae. Specifically, phosphorus concentrations exceeding 80 mg/kg were associated with a higher frequency of algae (*P* = 0.04). Interestingly, total phosphorus was the only determinant of site-specific species composition of green microalgae in harsh environments, such as coastal dunes (49).

Soils across various climatic zones exhibit significant differences, which are heavily influenced by temperature, precipitation, solar radiation, and seasonality. These climate components affect the biodiversity of microalgal populations (35). Poland is located within the temperate climate zone (50), where soils generally exhibit higher organic carbon (SOC, soil organic carbon) content (with an average range of 4–109 g/kg) (51) compared to tropical and subtropical climates (52). This is chiefly attributed to moderate temperatures and consistent precipitation (53), which promote the decomposition of plant material (54). Soils in temperate climates contain a relatively high humus content, whereas tropical soils are characterized by limited accumulation of organic matter due to their rapid decomposition rate and leaching (52, 54, 55). On average, the SOC content in native and cultivated sub-tropical soils ranges from 10.1 to 24.6 g/kg. Carbon turnover occurs at approximately double the rate in sub-tropical regions compared to temperate regions. This leads to a rapid depletion of organic matter, especially when soils are deprived of natural plant cover or designated for cultivation, and thus highly susceptible to erosion (54, 56).

In the present study, *Prototheca*-positive samples were isolated from soils classified as sandy and sandy-loam and, to a lesser extent, clay. For these soil texture types, the average SOC content has been estimated at 13 g/kg (56), which is close to that observed in our study (17.4 g/kg) and previously documented for the country (57). For the reasons mentioned above, soils in temperate climates have relatively high SOC contents, which should favor the growth of *Prototheca*. However, it is in tropical and subtropical regions that the algae have been isolated most frequently (15, 16). This may relate to a high water-holding capacity of the soil. Tropical environments experience very low diurnal and annual temperature fluctuations and constant high humidity (58). On the other hand, it has been demonstrated that water retention increases for soils with high SOC contents of all textural classes, especially for sandy soils (59). Another plausible link involves soils rich in iron oxides, commonly referred to as red soils, which predominantly occur in tropical and subtropical regions as a result of intense rock weathering (60). This observation aligns with documented cases of *Prototheca* algae isolation from soils in iron-rich environments, including Japan and Thailand (15, 16).

In Poland, red soils cover approximately 1% of the country’s surface and primarily develop on carbonate rock substrates found mainly in the Lesser Poland and Lublin Uplands (61). Notably, the latter exhibited the highest recovery rate of *Prototheca*, accounting for half of the isolates obtained in the study, further supporting a potential association between iron-enriched soils and the presence of the algae.

Altogether, it seems that it is not the climate *per se*, but rather the local combination of site-specific factors that influence the occurrence of *Prototheca* algae in the soil, as was shown for microalgae of the *Nannochloropsis* genus (62).

In conclusion, this study represents the first investigation into the occurrence of *Prototheca* spp. in soil ecosystems in Poland. Our findings suggest that the algae are scarcely present in soils under a temperate climate and should not be regarded as permanent terrestrial residents. This claim might be refuted with new findings from culture-independent approaches. Elevated phosphorus, higher pH, and salinity levels, along with anthropogenic activity and periodic flooding, are among factors that appear to favor the presence of *Prototheca* spp. in soils. The exceptionally low recovery of the algae challenges the prevailing paradigm of their widespread occurrence in soil ecosystems.

## Data Availability

All strain sequences were deposited in the GenBank database under the following accession numbers: PQ775131, PQ775132, PQ775133, PQ775134, PQ775135, PQ775136, PQ775137, PQ775138, PQ775139, PQ775140, PQ775141, PQ775142, and PQ775143. In addition, these sequences have been deposited in the Prototheca-ID database (https://prototheca-id.org/). Other data will be made available on request.
